# Mercury affects the phylloplane fungal community of blueberry leaves to a lesser extent than plant age

**DOI:** 10.1080/21501203.2017.1397063

**Published:** 2017-11-06

**Authors:** Katalin Malcolm, John Dighton, Tamar Barkay

**Affiliations:** a Graduate Program in Ecology and Evolution, Rutgers University, New Brunswick, NJ, USA; b Rutgers Pinelands Field Station, New Lisbon, NJ, USA; c Department of Biochemistry and Microbiology, Rutgers University, New Brunswick, NJ, USA

**Keywords:** Phylloplane fungi, mercury, heavy metal, community, pollution

## Abstract

Mercury (Hg) is a toxic heavy metal pollutant that is globally distributed due to atmospheric deposition to non-point source locations. Leaf surfaces directly sequester atmospheric Hg. Little is known of how phylloplane (leaf surface) fungi are influenced by Hg pollution. Through culture-based methodology, this study analysed fungal phylloplane community identity following a single-dose response to HgCl_2_ concentrations between 0 and 20 times ambient levels for New Jersey. Time passed following the Hg addition had a strong influence on the fungal phylloplane community, associated with natural successional changes. Mercury, however, did not significantly affect the phylloplane community identity. Notably, the control group was not significantly different than any of the Hg treatments. How the phylloplane functional group responds to Hg pollution has not been previously investigated and more research is needed to fully understand how Hg influences fungal phylloplane ecology.

## Introduction

Mercury (Hg) is a highly toxic heavy metal of biological and ecological concern. Although Hg is released naturally into the atmosphere (e.g. volcano emissions), nearly two-thirds of emissions are attributed to direct or indirect anthropogenic causes (Driscoll et al. 2007). Once Hg enters the atmosphere, it may circulate the globe over the course of its 1-year residence time (Schroeder and Munthe 1998). It is thus considered a global pollutant because it reaches areas remotely located from Hg sources through wet and dry deposition from the atmosphere (Fitzgerald et al. 1998; Morel et al. 1998; Selin 2009). In terrestrial systems, some of the deposited Hg interacts with vegetation and plants that serve as a sink for atmospheric Hg (Stamenkovic and Gustin 2009). The Hg found in leaves is atmospheric in origin, as translocation of Hg from soil to aboveground biomass is virtually non-existent (Ericksen et al. 2003; Gustin et al. 2008; Risch et al. 2012).

The phylloplane (leaf surfaces) supports a diverse community of microorganisms including bacteria, filamentous fungi, yeasts and protozoa (Meyer and Leveau 2012). The fungal epiphytic community is primarily dominated by Ascomycota and includes commensals, mutualists or facultative pathogens (e.g. Jumpponen and Jones 2009; Kembel and Mueller 2014). Phylloplane fungal communities can be influenced by environmental pollution. Mowll and Gadd (1985) observed a shift in the phylloplane community and an increase in *Aureobasidium pullulans*, at a lead contaminated site. Similarly, Joshi (2008) found that some phylloplane species (*Mortierella* spp., *Fusarium oxysporum* and *A. pullulans*) were more dominant in the community at a site with trace metal and gaseous contaminants as compared to the community from an unpolluted site. Other types of pollutants can influence the fungal phylloplane community, such as acid rain (e.g. sulphur dioxide) and increased ultraviolet radiation (Magan and McLeod 1991; Newsham et al. 1997). The leaf habitat and its inhabitants are involved in many ecosystem processes (such as nutrient cycling), yet the phylloplane is often underrepresented in ecological testing (Meyer and Leveau 2012).

It has not been determined how Hg pollution affects phylloplane fungi. However, it is established that fungi are capable of bioaccumulating Hg as well as possibly methylating Hg to its most toxic form (Fischer et al. 1995). Heavy metals can have an inhibitory effect on growth of fungal phylloplane species, although the degree of the effect appears to be species specific (Smith 1977). A change in the fungal community due to Hg exposure could have ecological consequences, such as a change in species interactions or alterations to leaf decomposition.

This study seeks to determine the effect of a single-dose application of HgCl_2_ on fungal phylloplane community of highbush blueberry leaves. Our aim in this study is not to push the system to known toxic levels or to test the effect of Hg on plant production. Many fungi can tolerate high concentrations of heavy metals, especially mycorrhizae (Orlowska et al. 2013), and endophytes (Shen et al. 2013) or as part of a microbial toolbox (Gadd 2004; Sprocati et al. 2014) targeted for bioremediation. Instead, we selected ambient and elevated Hg values to better understand the effects of Hg atmospheric deposition on the fungal phylloplane community of a more natural system. Hg sequestered in leaves is atmospheric in origin and deciduous plant leaves, like the blueberry plants used in this study, are exposed to atmospheric Hg for only a portion of the year before senescence and litterfall. Furthermore, it is well documented that the biogeochemical cycling of atmospherically deposited Hg (i.e. methylation and bioavailability in food chains) is delayed due to sequestration in decaying foliage and forest soil (Demers et al. 2007; Fu et al. 2010). It is therefore more appropriate to utilise concentrations of Hg that bracket ambient levels, instead of testing known toxic levels.

New Jersey receives 15 μg m^−2^yr^−1^ of Hg in the form of wet deposition (NJDEP 2002). In the NJ Pine Barrens, Hg contamination is atmospheric in origin as the site is not located near a point source of Hg pollution (Crane et al. 2012). Therefore, sequestration of Hg by plant foliage is likely a sink for the deposited mercury. We predict that the presence of Hg pollution, simulated through the application of ambient and elevated levels HgCl_2_, will affect the phylloplane fungi community.

## Materials and methods

### Experimental design

This study was conducted at the Rutgers Pinelands Field Station located in Brendan Byrne State Forest, NJ, USA, where blueberry (*Vaccinium* spp.) and huckleberry (*Gaylussacia* spp.) largely contribute to the forest understory (Conn and Dighton 2000). Wet deposition of Hg was simulated by applying known mass of HgCl_2_ to individual leaves of Pinelands native blueberry plants (*Vaccinium corymbosum*). In order to prevent runoff of solution, medium-sized leaves, that faced a perpendicular angle, were selected for study. The concentrations were determined by converting the NJ annual wet deposition of Hg value (15 μg Hg m^−2^yr^−1^) to a per leaf area basis. The experiment was conducted twice, in 2012 and 2013. For each year, an average leaf area (2012: 8.84 ± 1.85 cm^2^; 2013: 7.85 ± 2.15 cm^2^) was determined for the conversion. We approached this experiment by bracketing ambient and elevated Hg levels; in order to increase treatment groups, resulting in three replicate plants per year per treatment.

In the 2012 experiment, each plant was subjected to one of three treatments (*n* = 3). Treatments consisted of a 0× (control) in which only water was applied, a 1× ambient level Hg treatment (0.013 μg HgCl_2_ per leaf), and a 4× ambient level (0.053 μg HgCl_2_ per leaf). 50 μl of the HgCl_2_ solution was pipetted onto the adaxial surface of approximately 100 individual leaves, of similar surface area, per plant. The volume added was selected to avoid loss of the solution from the leaf. The Hg application occurred once in July 2012. The plants were maintained in a randomised block design in a polythene hoop greenhouse without temperature control.

In the 2013 experiment, each plant was subjected to one of four treatments (*n* = 3). Treatments consisted of a 0× (control) in which only water was applied, a 4× ambient level Hg treatment (0.047 μg HgCl_2_ per leaf), a 10× ambient level (0.118 μg HgCl_2_ per leaf) and a 20× ambient treatment (0.236 μg HgCl_2_ per leaf). Differences in amount of Hg added per leaf between years are due to differences in average leaf area in different years. 30 μl of the HgCl_2_ water solution was pipetted onto the adaxial surface of approximately 250 individual leaves, of similar surface area, per plant in June 2013. The experimental design and conditions were as above.

### Fungal isolation

In 2012, five leaves from each plant (*n* = 3) were removed 2, 14, 42 and 70 days after the Hg application. In 2013, 20 leaves from each plant (*n* = 3) were removed 3, 31, 59 and 87 days after the Hg application (henceforth referred to as time since Hg addition). The proportion of leaves were chosen in order to manage the total number of isolates, while still having enough leaf material to perform all of the analyses. A severe heat wave killed two plants and *n* = 2 for 0× and 4× treatments at days 31, 59 and 87. Immediately after leaves were harvested, leaves from each plant were composited (pseudoreplicates) and placed in sterile deionised water. They were placed in an orbital shaker for 1 hour at 170 RPM to remove surface spores and bacteria (Stanwood and Dighton 2011). Using sterile techniques, leaves were cut into quarters and randomly selected. Five leaf quarters from each plant were plated onto Potato Dextrose Agar (PDA, Carolina Biological, Burlington, NC) in 2012 and four leaf quarters were plated onto PDA in 2013. Petioles were removed before plating and in order to target epiphytic fungi on the leaf lamina, leaves were not surface sterilised (Osono 2007). The PDA plates were incubated at 22**°**C and fungal colonies were aseptically transferred to separate PDA plates to obtain pure fungal isolates. One culture method was chosen for standardisation to compare results across years, as different methodologies could potentially yield different results. Even with using molecular methods, there can be discrepancies between the communities identified depending on technique. Samarajeewa et al. (2015) reports bacterial communities identified by DGGE, RFLP and ion torrent clonal sequencing showing vast differences in communities revealed by each technique.

Each year, hundreds of isolates were cultured and the fungal isolates were separated into morphotype groupings. Morphotypes were determined by the physical appearance of the colony on the plate (e.g. colony shape, growth patterns, colour) and by microscopic analysis of spores when present. There were 84 morphotype groups and 98 morphotype groups in 2012 and 2013, respectively. Presence or absence of the morphotypes for each leaf harvest date and treatment were determined and converted to binary data.

### Molecular identification of fungal cultures

A representative plate from each morphotype was aseptically transferred to a PDA plate with cellophane on the surface to reduce risk of PDA contamination during molecular analysis. The samples were incubated at 22°C. The hyphae were harvested by removing the fungi from the cellophane and storing the sample in 1.5 ml cryogenic vials at −20°C. The majority of our cultures grew on the cellophane plates (96.4% in 2012 and 87.8% in 2013). For standardisation, the subsequent molecular analysis is of cultures that successfully grew on the cellophane plates.

DNA was extracted with the Powersoil**®** DNA Isolation Kit (MO BIO Laboratories, Inc. Carlsbad, CA). ITS (internal transcribed spacer) regions were amplified using primers ITS1F (CTTGGTCATTTAGAGGAAGTAA) and ITS4 (TCCTCCGCTTATTGATATGC) (O’Brien et al. 2005). In 2012, PCR reactions were conducted in 12 μl volumes (3 μl nuclease-free water, 6 μl GoTaq Polymerase, 1 μl of 10μM ITS1F, 1 μl of 10μM ITS4, 1 μl DNA template). In 2013 (to reduce chance of nonspecific bands or primer-dimers), PCR reactions were conducted in 12 μl volumes (4 μl nuclease free water, 6 μl GoTaq Polymerase, 0.5 μl of 10μM ITS1F, 0.5 μl of 10 μM ITS4, 1 μl DNA template). The PCR protocol used for both years was as follows: initial denaturation step of 94°C for 5 mins, followed by 32 cycles of 94°C for 30 s, 53°C for 30 s, 72°C for 1 min, with a final extension at 72°C for 10 mins.

In 2012, PCR products were cleaned with UltraClean**®** PCR Clean-Up Kit (MO BIO Laboratories, Inc. Carlsbad, CA). The cleaned PCR products were sent to Genewiz (South Plainfield, NJ) for sequencing. In 2013, PCR products were sent directly to Beckman-Coulter Labs for sequencing. The sequences were used as queries with the online Basic Local Alignment Search Tool (BLAST) of the nucleotide database at the National Center for Biotechnology Information (NCBI). Only sequences identified to at least a genus level with ≥98% identity matches were accepted. Sequences which were statistically significant (Table 2–3) were submitted to GenBank under accession numbers MG209662-MG209675. There were 25 genera molecularly identified in 2012, and 23 genera identified in 2013. Presence and absence of each genus for each leaf harvest date and treatment were determined and converted to binary data.

**Table 1.** Summary of PCA analyses of fungal phylloplane communities. Axis coordinates were analysed by MANOVA and mean separation by Tukey’s post hoc test.

Year	Identification	PCA Axis Contributions	Significance by time since Hg addition (MANOVA)	Significance by time since Hg addition (Tukey’s post- hoc, *p* < 0.05)	Significance by Hg treatment (MANOVA)
2012	Morphological	Axis 1 = 8.9% Axis 2–7.8%	Wilks’ Lambda value = 0.5560, F = 2.62, *p* = 0.0289	Axis 1: day 2 different than day 70	-
	Molecular	Axis 1 = 18.4% Axis 2–16.2%	Wilks’ Lambda value = 0.4259, F = 4.08, *p* = 0.0023	Axis 1: day 70 different than all other dates	-
2013	Morphological	Axis 1 = 11.0% Axis 2–8.3%	Wilks’ Lambda value = 0.1492, F = 13.24, *p* < 0.0001	Axis 1: all dates different from one another except day 31–59 Axis 2: day 3 different than day 87	-
	Molecular	Axis 1 = 15.2% Axis 2–14.1%	Wilks’ Lambda value = 0.3301, F = 6.17, *p* < 0.0001	Axis 1: day 31 different from all other dates Axis 2: day 3 different than day 59	-

**Table 2.** Significant (*p* < 0.05) morphotypes or genera by time since Hg addition and/or treatment when analysed by Chi^2^ in 2012.

Significant morphotypes or genera in 2012 study				
Morphological ID	Chi^2^ value	*p*-value	Significance by time since Hg addition	Significance by treatment
11 (yellow mycelia, small brown spores)	4.6170	0.0317	Occurred at 42d and 70d	-
25 (*Botryotinia* sp.) (white mycelia, dark sclerotia) Accession #: MG209662	6.8339	0.0089	Occurred mostly at 2d	-
Molecular ID	Chi^2^ value	*p*-value	Significance by time	Significance by treatment
*Fusarium* sp. Accession #: MG209663	12.7794	0.0004	Occurred mostly at 70d	-
*Nigrospora* sp. Accession #: MG209664	12.6191	0.0004	Occurred mostly at 70d	-
*Botryotinia* sp. (morphotype 25) Accession #: MG209662	4.2252	0.0398	Occurred mostly at 2d	-
*Penicillium* sp. Accession #: MG209665	12.5205	0.0019	Occurred mostly at 42 and 70d	
	6.9023	0.0086		Occurred mostly at 4×
*Cladosporium* sp. Accession #: MG209666	4.0798	0.0434		Occurred mostly at 4×
*Phoma* sp. Accession #: MG209667	13.6031	0.0002	Occurred mostly at 70d	

(Only significant morphotypes and genera are shown. In 2012 there were 84 morphotypes and 25 molecular genera.) Morphological ID includes corresponding molecular genus (if identified). Molecular ID name includes significant morphotype ID number.

**Table 3.** Significant (*p* < 0.05) morphotypes or genera by time since Hg addition and/or treatment when analysed by Chi^2^ in 2013.

Significant morphotypes or genera in 2013				
Morphological ID	Chi^2^ value	*p*-value	Significance by time since Hg addition	Significance by treatment
1 (*Alternaria* sp.) (brown mycelia, darker pigmentation appears to form rings) Accession #: MG209668	6.4184	0.0113	Occurred at all times, increased in frequency over time	-
3 (*Pestalotiopsis* sp.) (white-tan mycelia, spores present at centre of colony) Accession #: MG209669	10.0822	0.0015	Occurred mostly at 3d	
	16.2895	0.0003		Occurred mostly at 0× and 4×
4 (*Epicoccum* sp.) (red-tan thin mycelia, stained agar dark red) Accession #: MG209671	13.0244	0.0003	-	Occurred only at 0× and 4×
19 (*Epicoccum* sp.) red-tan thin mycelia, thicker masses of white mycelia, stained agar dark red) Accession #: MG209672	8.5102	0.0035	Occurred mostly at 3d	-
23 (thin white mycelia, formed thicker rings, dark spores)	12.7485	0.0004	Occurred mostly at 87d	-
27 (white-brown centre of colony, increased pigmentation at edge)	4.3594	0.0368	Occurred at all times, mostly at 59 and 87d	-
29 (*Epicoccum* sp.) (tan mycelia grew aerially to lid of plate) Accession #: MG209673	4.1922	0.0406	Occurred at all times, mostly at 87d	-
31 (*Pestalotiopsis* sp.) (white-brown mycelia with spots of dark brown) Accession #: MG209670	6.9545	0.0084	Occurred at 59 and 87d	-
48 (*Epicoccum* sp.) (tan-brown hyphae) Accession #: MG209674	7.1580	0.0075	Occurred mostly at 87d	-
Molecular ID	Chi^2^ value	*p*-value	Significance by time since Hg addition	Significance by treatment
*Alternaria* sp. (morphotype 1) Accession #: MG209668	6.4184	0.0113	Occurred at all times, increased in frequency over time	-
*Epicoccum* sp. (morphotypes 4,19, 29 & 48) Accession #s: MG209671-MG209674	6.4184	0.0113	Occurred at all times, increased in frequency over time	-
*Cladosporium* sp. Accession #: MG209675	7.9561	0.0048	Occurred Mostly at 3d	-

(Only significant morphotypes and genera are shown. In 2013 there were 98 morphotypes and 23 molecular genera.) Morphological ID includes corresponding molecular genus (if identified). Molecular ID name includes significant morphotype ID number.

### Total mercury (THg)

Five leaf quarters were acid digested overnight with trace metal grade 4HCl:1HNO_3_ and diluted at room temperature with 0.07N BrCl the next day (adapted from EPA Methods 1631B, 1630 and 7474). A subsample of the digestate was analysed by Cold Vapor Atomic Fluorescence Spectrometry (CVAFS, Brooks Rand Instruments Seattle, WA). Accuracy of total mercury (THg) values was confirmed through analysis of a standard reference material (NIST-SRM 1515 Apple leaves) and the detection limit was 0.06 μg/g. THg values were reported as μg/g dry leaf.

### Data analysis

Data from each year was analysed separately with binary presence/absence data for time since Hg addition and treatment. Data were analysed by Principal Component Analysis (PCA) using PC-Ord for morphotype groups and for molecularly identified groups. Separation on PCA axis coordinates were analysed by Multivariate Analysis of Variance (MANOVA) (Generalised linear model (GLM) for samples with uneven number of replicates) and mean separation was performed using Tukey’s HSD post-hoc tests (SAS 9.4 Cary, NC). Chi*^2^* analysis was performed on individual morphotypes and molecularly identified genera (SAS 9.4 Cary, NC). THg data were analysed by two-way ANOVA (GLM) to determine the effect of time since Hg addition and treatment. One-way ANOVA (GLM) was used to analyse THg at individual leaf harvest dates. Mean separation was performed using Tukey’s HSD post-hoc tests (SAS 9.4 Cary, NC).

## Results and discussion

**Figure 1.**
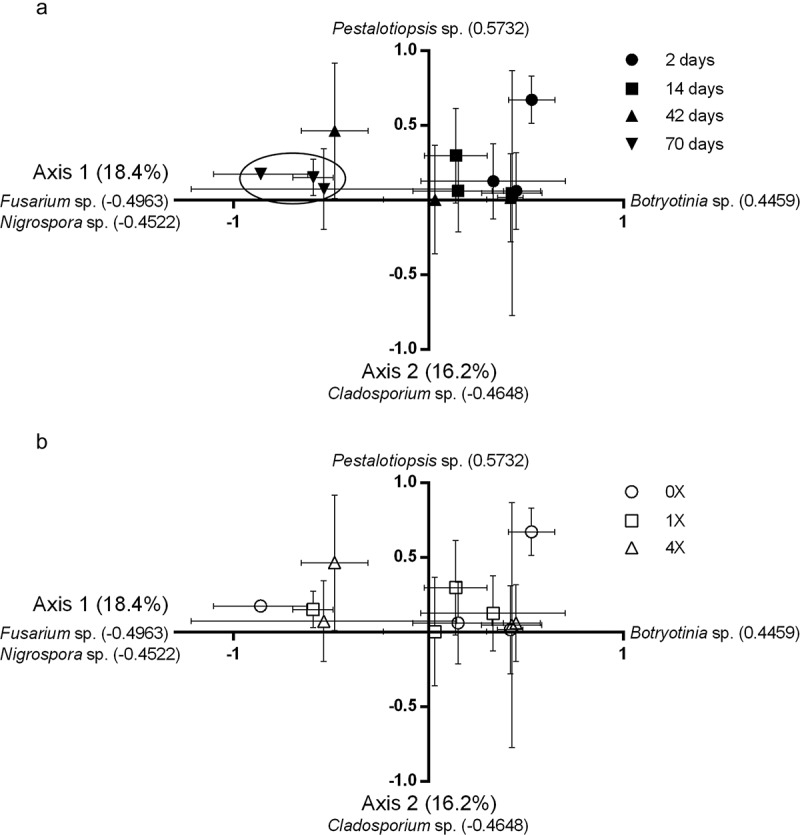
(a) PCA analysis of 2012 greenhouse experiment based on molecular identification of fungal cultures. Data points represent mean ± SE and day values represent time since Hg addition. When axis coordinates were analysed by MANOVA, there was a significant difference by time since Hg addition (Wilks’ Lambda value = 0.4259, F = 4.08, *p* = 0.0023). The Tukey test indicated that day 70 (circled) was significantly (*p* < 0.05) different from all other leaf harvest dates on axis 1. (b) PCA analysis of 2012 greenhouse experiment based on molecular identification of fungal cultures. Data points represent mean +/– SE based on Hg treatment. When axis coordinates were analysed by MANOVA, there was not a significant difference by treatment or time since Hg addition by treatment interaction.

The results of this preliminary study indicate that time since Hg addition is a more influencing factor on fungal phylloplane community identity than Hg treatment. Regardless of categorical system utilised (morphotypes or molecular genera) time since Hg addition significantly affected (*p* < 0.05) the fungal community identity in both years. Results of the PCA analyses for both years are reported in Table 1. PCA of the 2012 molecularly identified fungi in regards to time since Hg addition and Hg treatment are also graphically presented in Figure 1(a).

In 2012 (morphotypes and genera) and 2013 (morphotypes), the community on the first leaf harvest event was significantly (*p* < 0.05) different than that of the last harvest event (Table 1). This expected result is part of the natural successional patterns on the leaf (e.g. Lindsey and Pugh 1976; Wildman and Parkinson 1979). As the leaf ages, changes to substrate quality or competitive interactions could influence the community that can inhabit the leaf at a given time (Nix-Stohr et al. 2008; Osono 2008).

This trend is consistent with the exception of the 2013 molecular genera analysis (Table 1). The second leaf harvest date was significantly (*p* < 0.05) different than all other leaf harvest dates on axis 1. On axis 2, the first and third leaf harvest dates were significantly different. However, it is important to note that a severe heat wave occurred before the second leaf harvest date, killing two plants. Although the other plants survived, it is possible that increased heat and UV radiation altered the fungal communities and changed the dynamics for the remainder of the growing season (Newsham et al. 1997).

Changes in the phylloplane fungal community composition in both years are seen to be significantly influenced time since Hg addition, but have no significant interaction with mercury concentration added to leaves (Table 1).

When morphotypes or genera were analysed individually some were significantly (Chi^2^*p* < 0.05) different by time since Hg addition (Tables 2 and 3). There was one morphotype in 2012 (Table 2) and two morphotypes in 2013 (Table 3) that occurred earlier in the growing season of the plant. When analysed by genera, *Botryotinia* spp. (2012) (Table 2) and *Cladosporium* spp. (2013) (Table 3) were isolated more frequently earlier in the growing season of the plant and less frequently later in the growing season. *Botryotinia* spp. and *Cladosporium* spp. are considered common phylloplane saprophytes (Cambell and Clayton 1963; Hudson 1968).

There was one morphotype in 2012 (Table 2) and six morphotypes in 2013 (Table 3) that occurred more frequently later in the season. In 2012 (Table 2), *Fusarium* spp., *Nigrospora* spp., *Penicillium* spp. and *Phoma* spp. were isolated mainly in later leaf harvests. *Penicillium* spp. is a common phylloplane saprophyte (Nix-Stohr and Dighton 2004) and many *Fusarium* spp. are considered plant pathogens (Doohan et al. 2003). *Phoma* spp. contains pathogenic and saprophytic species (Boerema et al. 2004). *Nigrospora* spp. has been identified as pathogenic cause of leaf spot in *Vaccinium corymbosum* (blueberry plants) (Wright et al. 2008). The increase of pathogenic species over time since Hg addition is likely associated with changes of an ageing leaf. In 2013 (Table 3), common saprophytes *Alternaria* spp. and *Epicoccum* spp. were isolated later in time since Hg addition. This is in agreement with the results of Wildman and Parkinson (1979) who also observed colonisation of *Alternaria* spp. and *Epicoccum* spp. in relation to leaf maturation.

Hg treatment did not significantly influence the fungal communities (Table 1, Figure 1(b)). There was no significant treatment effect (morphotypes or genera) in 2012 or 2013. Our culture-based methods and subsequent molecular identification of cultures were, however, sensitive enough to detect statistical differences in time since Hg addition. Differences in time are expected, given known successional changes of fungal phylloplane communities. Due to the fact that the culture-dependent methods detected a difference by time since Hg addition and not by Hg treatment, we conclude that the Hg applied did not affect the fungal phylloplane community. Notably, the fungal community at the 0X treatment was not significantly different from any other treatment (1× to 20× ambient levels) in either year.

Morphotypes and genera were also analysed individually (Tables 2 and 3), to determine if any individual morphotype/genera were significantly affected by Hg and/or time after Hg addition. Some were significantly (*p* < 0.05) different by treatment. In 2012 (Table 2), *Penicillium* spp. and *Cladosporium* spp. were mainly isolated from the 4× treatment, which may be due to species-specific tolerances to and interactions with Hg. For example, the high propagule density strategy of *Penicillium* spp. may be a competitive advantage at this Hg level (Nix-Stohr et al. 2008). In 2013 (Table 3), there were two morphotypes that occurred mostly at the lower Hg treatments 0× and 4× and not at 10× or 20×. These are likely morphotypes with a low resistance or tolerance to Hg. There were no morphotypes in 2012 or molecularly identified genera in 2013 that showed a difference between Hg treatments. Although Hg had an effect on some individual species of fungi, their contribution to the community was not large enough to significantly alter the whole community composition with the addition of Hg.

The effect of time since Hg addition on the abundance of selected fungal species was much more important than the effect of mercury mass added to the leaves. Table 2 shows changes in abundance of fungal species appearance being linked to time since Hg addition in 7 cases, but only two species to Hg concentration. Similarly, in Table 3, 11 species are linked to time since Hg addition and only two are linked to Hg concentration. Despite relatively low levels of replication, we have seen patterns of changes in fungal communities and individual species with time since Hg addition. If Hg affected community composition to the same degree as time since Hg addition, it should have been detectable with our methods.

**Figure 2.**
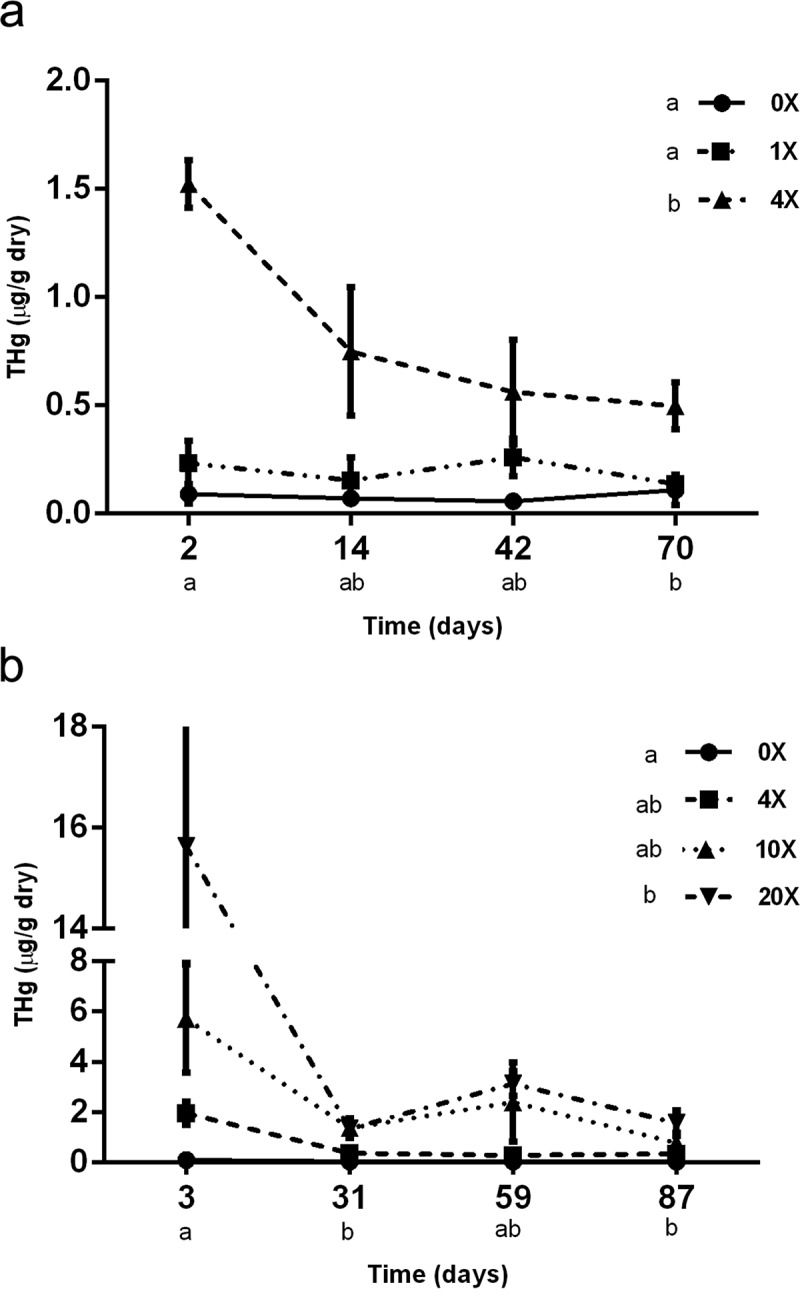
(a) Total Hg (μg/g) of dried leaves during the 2012 study. Data points represent mean +/– SE based on Hg treatment and day values represent time since Hg addition. There was a significant (*p* < 0.05) time since Hg addition and treatment interaction, time since Hg addition effect and treatment effect. Letters denote significant difference (*p* < 0.05) determined by Tukey’s test. (b) Total Hg (μg/g) of dried leaves during the 2013 study. Data points represent mean +/– SE based on Hg treatment and day values represent time since Hg addition. There were significant (*p* < 0.05) time since Hg addition and treatment effects. Letters denote significant difference (*p* < 0.05) determined by Tukey’s test.

THg in leaf tissue was significantly different (*p* < 0.05) by both time since Hg addition (F = 4.80, *p* = 0.0093) and treatment (F = 37.75, *p* < 0.0001) in 2012 (Figure 2(a)) and by time since Hg addition (F = 4.36, *p* = 0.0129) and treatment (F = 3.86, *p* = 0.0208) in 2013 (Figure 2(b)). There was also a significant time since Hg addition by treatment interaction (F = 4.14, *p* = 0.0054) in 2012 (Figure 2(a)) but not in 2013 (F = 1.74, *p* = 0.1309). In 2012, the 4× treatment had significantly higher (*p* < 0.05) THg levels than 0× and 1×. In 2013, the 20× treatment had significantly higher THg levels than 0× (*p* < 0.05) and 4× (*p* < 0.1).

Generally, THg in leaf tissue decreased over time since Hg addition and the higher Hg treatments had higher levels of THg than lower treatments. THg values are reported as μg per g of dry leaf and decreases in THg may therefore be the consequence of Hg loss as well as leaf growth. With leaf growth over time, the relative concentration of THg would appear to decrease by dilution as a result of the increasing leaf mass. It is also possible that a portion of Hg was emitted to the atmosphere, but this is likely to be minimal and expected to have occurred shortly following application to leaf surfaces (Stamenkovic and Gustin 2009). Indeed, the largest loss occurred immediately following the addition of mercury to leaf surfaces in both 2012 and 2013 (Figure 2). It is not known from our THg analyses if the Hg was retained within the leaf, on the leaf surface or associated with microfungi (Fischer et al. 1995; Stamenkovic and Gustin 2009; Crane et al. 2010) although other studies have suggested that Hg is largely retained in stomatal cavities and thus internal to the leaf surface (Demers et al. 2013).

Instead, these data suggest that a portion of the Hg was retained throughout the growing season of the blueberry plants and was potentially an influencing factor on phylloplane fungi. However, in 2012, the highest Hg concentration (4×) applied decreased by approximately 50% after 14 days and in 2013 the highest Hg concentration (20×) exhibited a tenfold decrease after 30 days. Thus, it is likely that a negative effect of Hg on fungi, if present, was transient and did not have a strong influence on the fungal community throughout the growing season of the plants.

The Pinelands site is not near a known point source of Hg and is considered a low contamination site largely due to atmospheric deposition of Hg. The results indicate that time following the Hg addition was a more influencing factor on the fungal phylloplane community than Hg treatment. The addition of Hg occurred in a single-dose application in levels equal to or exceeding annual ambient values in New Jersey. This suggests that the fungal community of the blueberry phylloplane is resilient to Hg pollution at levels that simulate atmospheric deposition and even those at several fold higher that ambient. There may be differences in Hg resistance or tolerance of different fungal functional groups to Hg, as ectomycorrhizal communities have been shown to be susceptible to Hg exposure (Crane et al. 2012). Phylloplane fungi are adapted to the stressed and fluctuating leaf habitat, for example melanin production protects against UV radiation (Magan 2007). It is of interest to further explore if adaptation to a stressed environment confers tolerance/resistance to Hg pollution for phylloplane fungi.

The phylloplane is a relatively understudied habitat system (Meyer and Leveau 2012) and an important interface that facilitates Hg transport from the atmosphere to terrestrial environments (Ariya et al. 2015). It is thus clear that more research is needed to investigate the interaction between Hg and phylloplane communities. This includes but is certainly not limited to, identifying community species and testing ecological theory. There is additional culture-based work that should be conducted, for example testing different Hg concentrations and different plant species. Altering lab environment conditions (e.g. media) for fungal cultures, as well as testing in a natural setting may also provide further insight. Future investigations would also benefit from the utilisation of additional molecular technologies such as high-throughput sequencing or community fingerprinting to allow detection of more subtle effects. Such technologies could identify rare species and quantify species abundance. Hg is a toxic heavy metal of ecological and biological concern. Additional research on the interaction between Hg and phylloplane fungi would meaningfully contribute to current understanding of, and public policies related to, the Hg cycle, heavy metal pollutants and fungal ecology.
